# Supplementary material for: Critical Cooperation Range to Improve Spatial Network Robustness
Source: PLoS One. 2015 Mar 20;10(3):e0118635. doi: 10.1371/journal.pone.0118635 (PMC4391338; doi:10.1371/journal.pone.0118635)
Supplement: S1 Table — (PDF) [file pone.0118635.s001.pdf]

TABLE - THE WORLD AIR-TRANSPORTATION NETWORK

The network that represents the WAN was carefully assembled in Ref. **[21]**, using data from OpenFlights (http://sourceforge.net/p/openflights/code/682/tree/openflights/data/, files airports.dat and routes.dat) and The World Bank dataset sourced through Civil Aviation Statistics of the World and ICAO staff estimates (http://data.worldbank.org/indicator/IS.AIR.PSGR), all for the year 2011. After removing redundancies and extrapolating some passengers information (Ref. [**21**]), we construct a network of 3237 airports and 18.125 flights (csv files available as SI).

Information about continents, contained in the original data set, was used to further break it down. Continental networks contain flights in which both end-points are in the same continent. Australia includes all islands in the Pacific ocean. For simplification, Russia is entirely part of Europe, and Turkey is entirely in Asia. A summary of the main characteristics of the continents and the WAN is present in the table below.

TABLE S1. Data of WAN and its continental components.

Name	Nodes	Links	Passengers (daily)	Flights (daily)	Average Degree	Average Passengers (daily)	Average Flights (daily)	Average Distance btw Airports (km)	Average Flight Distance (km)
Africa	269	642	220,481	3,023	4.77 ± 0.43	819.63 ± 129.59	2.35 ± 0.10	$3,759.92 \pm 10.52$	$1,139.23 \pm 44.35$
Asia	773	3,911	2,409,160	23,406	10.12 ± 0.64	$3,116.64 \pm 281.14$	2.99 ± 0.07	$4,089.57 \pm 4.16$	$1,338.13 \pm 19.76$
Australia	288	567	178,447	2,499	3.94 ± 0.39	619.61 ± 132.87	2.20 ± 0.11	$3,450.12 \pm 10.14$	810.18 ± 39.03
Europe	602	5,188	1,907,980	25,587	17.24 ± 1.07	$3,169.40 \pm 391.04$	2.47 ± 0.07	$2,410.24 \pm 4.05$	$1,250.84 \pm 12.11$
North America	1,006	4,289	2,344,470	20,593	8.53 ± 0.62	$2,330.48 \pm 245.49$	2.40 ± 0.08	$3,386.44 \pm 2.66$	$1,150.44 \pm 15.67$
South America	299	762	380,348	3,984	5.10 ± 0.44	$1,272.07 \pm 154.33$	2.61 ± 0.10	$2,719.93 \pm 6.84$	793.98 ± 27.81
World	3,237	18,125	7,440,880	94,644	11.20 ± 0.42	$2,298.70 \pm 127.80$	2.61 ± 0.04	$8,678.58 \pm 1.92$	$1,734.64 \pm 14.58$
